# Environmental Sources of Possible Associated Pathogens and Contaminants of Stingless Bees in the Neotropics

**DOI:** 10.3390/insects16040350

**Published:** 2025-03-27

**Authors:** Joseline Sofía Ocaña-Cabrera, Sarah Martin-Solano, Claude Saegerman

**Affiliations:** 1 Research Unit of Epidemiology and Risk Analysis Applied to Veterinary Sciences (UREAR-ULiège), Fundamental and Applied Research for Animal and Health (FARAH) Center, Faculty of Veterinary Medicine, University of Liège, Quartier Vallée 2, Avenue de Cureghem 6, B43a, Sart-Tilman, 4000 Liege, Belgium; jocana@doct.uliege.be; 2 Grupo de Investigación en Sanidad Animal y Humana (GISAH), Carrera de Ingeniería en Biotecnología, Departamento de Ciencias de la Vida y de la Agricultura, Universidad de las Fuerzas Armadas ESPE, Av. Gral. Rumiñahui S/N, Sangolquí 171103, Ecuador; ssmartin@espe.edu.ec

**Keywords:** Meliponini, threat, agrochemicals, disease agents, preventive measures, One Health

## Abstract

Stingless bees are crucial for pollination and support diverse ecological relationships, offering economic benefits and contributing to enhanced crop yields. Their tropical pollinator status makes them highly sensitive to environmental changes and disruptions, which could affect their survival, as well as to pathogens that threaten their health. The lack of comprehensive research and the scattering of reports make it difficult to identify pathogens and contaminants. This review aims to provide an overview of diseases in stingless bees, examine chemical contaminants in their products, and explore threatened sources. Using the PRISMA flowchart, a total of 30 articles from 2009 to 2024 concerning pathogens and contaminants in stingless bees were retrieved. A total of 15 pathogens and 26 pollutants affect life expectancy and survival rate of stingless bees (mainly the genera *Melipona* and *Tetragonisca*) were identified in five major areas of the Neotropics, including Brazil, Mexico, Costa Rica, Australia, and Asia. Studies indicated that the bacterial genera *Pseudomonas*, *Melissococcus*, and *Lysinibacillus* are affecting the survival of stingless bees, particularly their brood, and contributing to annual colony deaths. Heavy metals, polycyclic aromatic hydrocarbons (PAHs), and microplastics have been detected in by-products of stingless bees, especially honey. Epidemiological research is crucial, including studies on pathogens associated with diseases, the effects of contaminants on bees, and the development of quality guidelines for stingless-bee products.

## Simple Summary

The Meliponini tribe of bees, which are distributed in tropical and subtropical climates around the world, play an important role in pollination. It is imperative to ascertain the microorganisms and contaminants that impact them, which may also be of human origin, to implement preventive measures for their conservation. A comprehensive investigation was conducted into agents associated with stingless-bee diseases and contaminants, as well as their origin and spread. The presence of bacteria and viruses associated with a particular syndrome that results in the death of colonies of the *Melipona* species has been identified. Contaminants found in materials inside the nest, as well as in the products derived from stingless bees and destined for human consumption, were indicative of the quality and health of the environment surrounding the nests, increasing the vulnerability of the bees. It is imperative to expand research efforts to explore the health of bees in greater depth from a One Health perspective and to elucidate how biotic and abiotic factors pose threats to the lives of stingless bees, both individually and in combination with other factors.

## 1. Introduction

Stingless bees (Hymenoptera, Apidae, Meliponini) are a large and ecologically vital group of eusocial bees, with over 600 species, predominantly found in tropical regions [1]. They play an important role in the environment by affecting how plants reproduce and supporting different relationships between plants and insects [2,3]. Stingless bees also help with pollination, which can increase crop yields by almost 40% [4]. This makes them important for the economy. Stingless bees produce honey that has special medicinal properties [5], such as antimicrobial and antioxidant benefits [6]. This honey fights off germs and protects cells; it is valuable in the food, pharmaceutical, and cosmetic industries [7,8].

Stingless bees exhibit significant ecological and behavioral diversity [9], with different species showing various foraging strategies [10], colony sizes, and nesting behaviors [11]. The production and management of stingless-bee products, including honey and cerumen, have traditionally been part of local economies, particularly in tropical regions like Latin America and Asia [12,13,14,15] where specific species are cultivated for honey production. As interest in stingless-bee cultivation (meliponiculture) grows, the industry supports biodiversity conservation and offers a sustainable source of income for stingless-bee keepers, empowering rural communities. The economic and environmental importance of stingless bees underscores their potential as a cornerstone for both ecological preservation [16,17] and socio-economic development in tropical countries.

Stingless bees play a crucial role in pollination and in maintaining biodiversity in tropical and subtropical ecosystems [10]. Their foraging behavior and diet breadths are closely tied to forest cover [18], with species richness being higher near forest edges [16]. However, deforestation and habitat fragmentation threaten their persistence, as smaller species are particularly vulnerable [18]. Climate change poses additional risks [19,20], disrupting their developmental cycles, their social behaviors [21], and overall survival [22,23,24], which can further jeopardize ecosystems. As tropical pollinators, stingless bees are highly sensitive to environmental changes, making their conservation vital for maintaining biodiversity and ecosystem services in a warming world.

Factors like scent influence stingless-bee foraging behavior [25], along with the color [26], location, and temperature of flowers [27]. While they prefer feeders closer to the nest based on scent, their color preferences appear random [28]. Foraging decisions are also socially driven; returning bees share olfactory and gustatory information with nestmates [29], influencing future food choices. Additionally, many stingless-bee species rely on plant resins for nest building and defense. Species with a higher resin intake tend to be more active [30], highlighting the critical role of resins in their behavior and nest maintenance [31].

A major threat to the well-being of stingless-bee colonies is the transmission of parasites from other insects that interact with them while collecting food and materials from the same plants in the field [32]. Deformed wing virus (DWV) is currently among the most widespread insect pathogens on the planet, and its propagation has been linked to infestations of *Varroa* mites in honey bees [33]. The virus was classified into three distinct genotypes: DWV-A, DWV-B, and DWV-C. The latter has been identified as the most prevalent in the stingless bee *M. subnitida* in Brazil [34], and co-infections involving multiple genotypes, and the black queen cell virus has also been documented [35,36].

Bacterial strains of *Lysinibacillus sphaericus* [37], as well as fungal pathogens such as *Melissococcus plutonius* [38] and *Nosema ceranae* [39], have also been reported in stingless bees. The health implications of nests infected with these pathogens encompass various adverse outcomes, including brood mortality, diminished worker population [40], and the repercussions of Colony Collapse Disorder (CCD) [41], which can be exacerbated by pesticide exposure.

The increasing demand for land for monoculture crops, combined with unsustainable agricultural practices, has led to the degradation of natural habitats. This phenomenon is primarily attributable to the elevated demand for pesticides, which exert a direct impact on bees and other pollinators [42,43]. The direct effects of pesticides on stingless-bee species are size- and species-dependent, owing to the presence of specific detoxification mechanisms [44]. For instance, in *Melipona scutellaris*, alterations in the foraging-bee ascent rate and changes in heterochromatin were observed after topical exposure to fipronil [45].

Exposure to agrochemicals not only affects individual bees but also extends to products derived from the nest. In a region of Italy, an analysis of honey bees’ bee bread revealed the presence of 63 different pesticide residues, some of which were detected at levels that exceeded the risk threshold established for bees [46]. However, expanding pesticide risk assessment studies to non-*Apis* bee species remains a contentious issue among researchers [44,47,48], primarily due to differences in species biology. A significant gap in the existing body of toxicological research on stingless bees is highlighted by the limited number of studies conducted in Latin America. Notably, more than 80% of the published research focuses on Brazilian species, underscoring the paucity of studies on other species and the limited evaluation of crop pest products [49].

The characterization of contaminants and pathogens impacting stingless bees constitutes the initial step in determining the direction of priority research and identifying knowledge gaps. The study of pesticide contamination of stingless-bee products is particularly important to conservation efforts and sustainable agricultural practices. A comprehensive understanding of these impacts is essential for fostering improved land-management practices and more-sustainable agricultural techniques, which in turn can positively impact local economies as well as the domains of agriculture and stingless-bee keeping or meliponiculture. This systematic review has two main objectives: (1) to analyze diseases associated with pathogens in stingless bees and (2) to examine chemical contaminants present in their products. Additionally, recommendations based on the One Health approach will be proposed to mitigate these impacts.

## 2. Materials and Methods

The literature review on environmental contaminants affecting stingless bees in the Neotropics was conducted from 1 to 28 November 2024 under the Preferred Reporting Items for Systematic Reviews and Meta-Analysis for Scoping Reviews (PRISMA-ScR) 2018 checklist and the PRISMA 2020 flowchart.

A search was conducted in the Google Scholar and PubMed databases, using the following keywords and Boolean operators to find indexed articles: stingless bees AND Brazil OR Mexico OR Costa Rica OR Australia OR Asia, AND honey OR cerumen OR resins OR propolis, AND contaminant OR heavy metals OR neonicotinoids OR pesticides OR pathogens OR disease. These terms were used to retrieve all relevant publications, regardless of their year of publication. The selection of countries was based on their significant role in meliponiculture research and development within the Neotropics.

Exclusion criteria included the following: (i) language other than English, Portuguese, or Spanish, (ii) a focus on beekeepers, honey bees, wasps, and other Hymenoptera, (iii) duplicates between the two databases, (iv) information that is exclusively concerned with the methodology or the development of new methodologies, (v) toxicity studies, or (vi) literature of a comprehensive and overarching nature.

The data from the selected articles were compiled into a database to assess sampling efforts, measured by the number of publications. The database included records of contaminants in stingless-bee products from Neotropical countries (sample numbers and prevalence), the most frequently monitored native bee species, and habitat types (primary forest, secondary forest, disturbed areas, unspecified locations, urban areas, and agricultural zones).

## 3. Results

**Figure 1:**
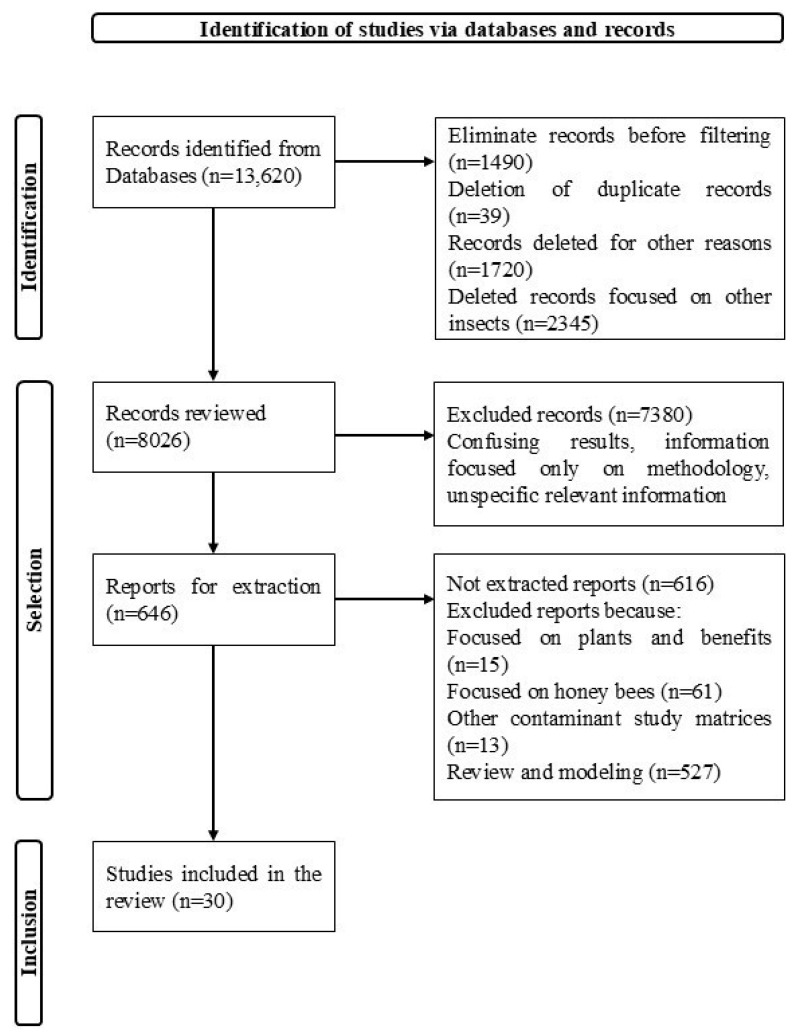
PRISMA 2020 flowchart.

This systematic review was conducted following the PRISMA guidelines (see Figure 1), allowing for the identification of pathogens (n = 15) and contaminants (n = 26) affecting stingless-bee survival in five selected primary areas representative of the Neotropics. The sampling effort (n = 30) covered key regions, including Brazil (n = 21), Mexico (n = 2), Costa Rica (n = 1), Australia (n = 4), and tropical and subtropical Asia (n = 2).

Studies conducted in geographically distinct regions—Australia, Brazil, and Mexico—reported the presence of pathogens and contaminants in various genera and species of stingless bees, as well as in diverse nest by-products such as honey, pollen, geopropolis, wax, and brood. In the tropical zone of the western hemisphere, Brazil had the highest number of studies reporting pathogens and pollutants. In contrast, Australia had the highest number of studies in the eastern hemisphere.

Diseases in stingless-bee brood of bacterial origin have been reported from distant areas such as Brazil and Australia, although different bacterial species have been reported in each case. While diseases associated with viruses have been reported exclusively in Brazil, this may be attributable to the level of research conducted in that country, without excluding the possibility of detection in other areas of Latin America where stingless bees are distributed. Concerning contaminants, Mexican reports have indicated the presence of trace amounts of pesticides, while Brazilian reports primarily concern trace metals in stingless-bee products, as do Australian reports.

### 3.1. Study Matrices for Pathogens and Pollutants in Stingless Bees

Adult foraging bees constituted the main sample (59%, 7/13) in detecting seven of the eight pathogen-associated diseases listed in Table 1. In certain studies, these individual bees were used together with honey (15%, 2/13), brood (7%, 1/13), worker and queen larvae (7%, 1/13), brood cell provisions (7%, 1/13), and pollen (7%, 1/13) from several stingless-bee species.

The species belonging to the genera *Melipona* (58%, 15/26) and *Tetragonisca* (15%, 4/26) have attracted the most interest. The stingless-bee species included in the studies are listed in order of increasing to decreasing presence: *Melipona quadrifasciata*, *Melipona marginata*, *Melipona rufiventris*, *Melipona mandacaia*, *Tetragonisca angustula*, *Tetragonisca fiebrigi*, *Scaptotrigona jujuyensis*, *Frieseomelitta varia*, *Trigona spinipes*, *Nannotrigona testaceicornis*, *Tetragona elongata*, and finally, two Australian stingless-bee species: *Tetragonula carbonaria* and *Austroplebeia australis*.

Most samples were obtained from domesticated or managed nests (88%, 7/8), while only one study reported sampling from both managed and wild nests. Furthermore, the predominant sampling season in pathogen-associated-disease studies was summer–spring, with only one study sampling during autumn–winter.

The matrices used for detecting contaminants (Table 2) in stingless-bee nests included honey (46%, 6/13), geopropolis (15%, 2/13), pollen (15%, 2/13), wax (7%, 1/13), individual bees (7%, 1/13), and larvae midguts (7%, 1/13). Most samples were obtained from domesticated nests (77%, 7/9), while one study was developed under laboratory conditions (bioassay). The predominant season for sampling was summer, and the focus was on long-term sampling, ranging from one to four years in duration.

The *Melipona* (38%, 8/21), *Scaptotrigona* (28%, 6/21), and *Tetragonisca* (19%, 4/21) species were the most prevalent genera in contaminant studies. The following species were involved in contaminant studies: *M. scutellaris*, *M. quadrifasciata*, *M. marginata*, *M. bicolor*, *S. bipunctata*, *S. postica*, *S. mexicana*, *Tetragonisca weyrauchi*, *T. angustula*, *Partamona helleri*, and one Australian representative species: *T. carbonaria*.

### 3.2. Bacterial, Fungi, and Viral Pathogens of Stingless Bees

Four diseases associated with bacteria were reported: Unknown annual colony collapse syndrome Disorder, European foulbrood, unknown annual syndrome (Brazil), and bacterial brood disease (Australia). The following groups of bacteria were attributed to stingless-bee or nest damage: Firmicutes Group U and Group Z, and Acetobacteraceae. The bacterial genera mentioned were *Pseudomonas* sp. and *Sphingomonas* sp., while the bacterial species identified were *M. plutonius*, *L. sphaericus*, *Escherichia coli*, and *Alcaligenes faecalis.* The stingless-bee species in which the most prevalent reports of diseases related to bacteria were documented were *Melipona* in regions of Brazil and *Tetragonula* in Australia.

The only microorganism in the fungal kingdom was *N. ceranae*, which causes nosemosis. The viruses reported were deformed wing virus (DWV) variants A and C, tailed viruses (Caudoviricetes), acute bee paralysis virus (APBV), and black queen cell virus (BQCV). The only two pathogens reported in two studies were *N. ceranae* and deformed wing virus. The genus *Melipona* had the highest number of reports of fungi- and virus-associated diseases in Brazil, followed by the genera *Tetragonisca*, *Scaptotrigona*, *Nanotrigona*, and *Tetragona*.

The prevalences among samples positive for pathogens were estimated to range from 21 to 87% of the total number of samples examined in each study (see Table 1). The pathogen with the highest percentage prevalence was *N. ceranae*, a microsporidium that causes a disease known as nosemosis. A case of co-infection was reported in a brooding sample of *M. marginata*, in Brazil, where the microsporidium *N. ceranae* and the bacterium *M. plutonius* together caused European foulbrood.

### 3.3. Anthropogenic Contaminants in Stingless-Bee By-Products

The following metals have been identified in various stingless-bee nest matrices from specific regions of Brazil and Australia. Notably, in all studies, these metals exceeded the detection and quantification limits of each laboratory: aluminum (Al), arsenic (As), calcium (Ca), cadmium (Cd), chromium (Cr), copper (Cu), iron (Fe), indium (In), magnesium (Mg), manganese (Mn), molybdenum (Mo), nickel (Ni), lead (Pb), antimony (Sb), tin (Sn), and zinc (Zn), as well as nanoparticles of titanium dioxide (TiO_2_). Honey, geopropolis, wax, and bees were presented as the main matrices for detecting trace amounts of these metals. The metals that were found to be of most concern were arsenic and lead in the honey samples, and aluminum and chromium in the geopropolis samples.

Compounds belonging to the organochlorine pesticide group—including heptachlor, endrin, gamma-hexachlorocyclohexane (γ-HCH), dichlorodiphenyltrichloroethane (DDT), and dichlorodiphenyldichloroethylene (DDE)—were also reported to be the most concerning contaminants, in honey and pollen from *Scaptotrigona mexicana* in Mexico.

Compounds derived from the oil industry or the burning of organic matter the polycyclic aromatic hydrocarbons, were reported in honey from the *Melipona*, *Scaptotrigona*, and *Tetragonisca* genera of stingless bees in Brazil. The compounds identified included light polycyclic aromatic hydrocarbons (PAHs), such as fluorene, phenanthrene, anthracene, and heavy PAHs, including benzo[a]anthracene, benzo[b]fluoranthene, benzo[k]fluoranthene, indeno[1,2,3-cd]pyrene, and dibenz[a,h]anthracene.

The presence of plastic microparticles of polystyrene (PS), polyethylene terephthalate (PET), and polypropylene was identified in honey from *M. quadrifasciata* and in the midguts of *P. helleri* larvae—both of which are Brazilian stingless-bee species.

## 4. Discussion

### 4.1. Occurrence and Reporting of Pathogens in Stingless Bees

*Nosema ceranae*, an epidemiologically significant parasite of honey bees [63], possesses spores that are highly resistant and spread via the oral–fecal cycle [64]. Cross-contamination in nature is likely due to the overlap in spatial distribution, range, and feeding resources among insects [65]. Specifically, within the order Hymenoptera, the transmission of this pathogen can occur via flowers through shared used by pollinators [66], but pathogens can also be transmitted through behaviors such as the theft of honey and pollen [67], the usurpation of nest sites, and the dispersal of spores by certain insectivorous birds [68]. These events facilitate the host-hopping of the pathogen and the subsequent territorial spread of the disease.

In the Argentine province of Misiones, colonies of *T. fiebrigi* and *S. jujuyensis*, which were positive for *N. ceranae*, were located near honey-bee hives, suggesting inter-species contact due to robbing behavior. In contrast, *N.-ceranae*-positive colonies in Argentina’s Chaco province were farther from managed honey-bee hives, indicating another form of contact with the spores [39].

In southeastern Brazil, the bacterium *M. plutonius* and symptoms of European foulbrood (EFB) were reported for the first time in *Melipona* species [38]. In areas where beekeeping and meliponiculture coexist, managed honey bees (*Apis mellifera*) and stingless bees (Meliponini) likely share plant resources, increasing the risk of pathogen and parasite transmission [69]. Furthermore, the notion that certain beekeeping practices can be extrapolated to meliponiculture has emerged as a potentially hazardous approach, such as the utilization of *A. mellifera* supplements for the feeding of stingless-bee colonies [70]. *Melissococcus plutonius* can modify its physiological state to ensure its survival [71]. This ability may represent a significant adaptive trait, enabling it to survive on honey-bee products and infect stingless-bee broods. It is important to note that the increased prevalence of EFB symptoms in Brazilian stingless bees can be attributed to elevated environmental stress factors, including a reduction in natural foraging habitat [24], and increased exposure to chemicals [47]. The effects of the EFB on unsealed stingless-bee larvae manifest in symptoms of illness, and a subsequent elimination due to hygienic behavior and worker inspection, indicative of social immunity [40]. In the case of *M. scutellaris*, colonies experienced a rapid collapse, followed by a phorid attack.

Even though not all stingless-bee pathogens are transmitted from honey bees, the bacterium *Lysinibacillus sphaericus* has been reported in two endemic stingless-bee species in Australia. This has resulted in a reduction in colony populations and a failure of the workers to maintain hive structures, which has had a direct effect on brood rearing [37].

Virus families such as Dicistroviridae, Parvoviridae, and Circoviridae have been reported in diseased stingless bees [72]. Nevertheless, there is still a considerable gap in investigating viruses associated with these eusocial bees. Metagenomic studies of honey bees have identified viruses originally described in plants, a subgroup of Aphid Lethal Paralysis Virus (ALPV), Israel Acute Paralysis Virus (IAPV), and Lake Sinai Virus (LSV) [73]. Initially, researchers hypothesized that these viruses were only environmental contaminants introduced into the nest through pollen and nectar collected by bees. However, subsequent research has revealed that these viruses may be utilizing the bees as hosts, as evidenced by the example of tobacco ringspot virus (TRSV), which, in addition to infecting *Apis mellifera*, has also been found to replicate in this bee species [74].

A holistic approach is recommended for studying viral communities in managed and wild bee species, including their food plants. Additionally, considering geographical context can provide deeper insights into bee–virus–plant interactions [75].

Viruses were present in the following products as well: BQCV in the bee pollen of *A. mellifera* and a protein-based commercial ration (prepared with the same *Apis mellifera* bee-pollen from the South of Brazil), and ABPV in the powder of *A. mellifera* bee-pollen (purchased from the Northeast of the Country) [76].

DWV-A and BQCV have been detected in two stingless-bee species in Mexico, with prevalences of 1% and 15%, respectively. In *M. beecheii*, experimental inoculation of pupae and adults with RNA viruses showed negative effects on colonies [69]. In the case of *M. colimana*, both viruses were found naturally in adult bees and replication of these viruses was demonstrated [36], making this species a possible host and vector for both viruses.

### 4.2. Bees and Nest By-Products as Bioindicators of Environmental Health

The use of honey-bee hives to study environmental pollutants has revealed that the bees themselves provide a more accurate reflection of environmental health than hive products [77]. In the case of stingless bees, the Australian species *T. carbonaria*, with a foraging range of 0.3–0.7 km [78], has been studied as a small-scale bio-indicator of trace elements in different landscapes [58]. The influence of *M. quadrifasciata*, *M. scutellaris*, and *T. bhingami* on metal accumulation in their geopropolis, particularly lead, has been documented [77].

Several studies have used bee products to detect and quantify contaminants. Propolis, pollen, and wax are more suitable for studying toxic metals than honey. As is illustrated in the present systematic review, more studies on metal detection utilized stingless-bee honey compared to other products. In instances where the objective was to evaluate the quality of the environment and nest health, bees emerged as the optimal study matrix. Conversely, if the concern pertains to food safety, the utilization of bee nest products was advocated, given their designated purpose for human consumption.

The presence of heavy metals, including lead (Pb) and cadmium (Cd), has been documented in trace amounts in bee specimens as well as in other matrices such as propolis, pollen, and honey [79,80,81]. These metals in the nest result from various factors, including the environmental quality of worker-bee foraging areas. In meliponiculture, nest-product harvesting avoids using steel utensils that could release traces of Al, Zn, or Fe. The honey or pollen pots are crafted from cerumen, a malleable (resins plus wax) material that opens without force to facilitate the collection of honey or pollen. In contrast, tools such as crowbars or spatulas are employed to access the nest, given that batumen, a comparatively rigid material, or propolis, in certain stingless-bee species, occasionally necessitates the application of force or support to displace the floors of the technical nest.

If the species of pollen and nectar plants are exposed to chemical contaminants, the final composition of the honey and bee pollen produced in those locations may contain contaminating elements [80]. Other anthropogenic sources of environmental metals include vehicle emissions [82,83], mining- and industrial-waste smelting [84,85], and residual leaded petrol, which persists in the environment [86,87].

Iron (Fe) and aluminum (Al) are among the most prevalent elements in the Earth’s crust [88]; however, concerns arise when these elements are found in concentrations exceeding 20 and 2 mg/kg, respectively [89,90]. These concentrations represent the permissible limits for these elements in food. While there is no global regulation for products derived from stingless bees intended for human use or consumption, the presence of concentrations above the reference concentrations in honey or geopropolis suggests a potential food safety concern.

In the context of the bumblebee area, the bees were exposed to arsenic oxide, cadmium chloride, or chromium oxide in a sucrose solution. The results indicated that significant amounts of cadmium (CdCl_2_ 10.3 mg/L) were accumulated in the bodies of the exposed bees. However, no accumulation of chromium or arsenic was observed [91]. While it is improbable that foraging bumblebees or other bees will encounter lethal concentrations of these metals in the field, it is imperative to comprehend how sublethal concentrations influence colony functionality, given the observed variation in bee responses to different metal species. As an example, laboratory experiments with worker bees (*Apis cerana cerana*) demonstrated that chronic exposure to low-level concentrations of Cd resulted in a significant reduction in the number of antioxidant gene transcripts. Additionally, Cd inhibited the transcription of immune-related genes and altered the structural characteristics of bacterial and fungal communities within the bee gut [92].

Pesticides and heavy metals have been shown to induce changes in the composition of the microbiome, cellular damage in the midgut tissue, and a disruption of the peritrophic membrane in honey bees [93]. The latter physiological effects may increase the susceptibility of social insects to intestinal or bacterial pathogens. Conversely, the impact of plastic microparticles has been associated with a decline in intestinal microbiota, modifications in the expression of genes associated with oxidative damage and detoxification, and alterations in the cognition and nervous system of honey bees [94].

In the case of stingless bees and other contaminants, analytical investigations were conducted in Brazil on *M. subnitida* honey samples from urban and rural areas. The analysis yielded positive results for the presence of organophosphorus compounds. Subsequent comparative analyses of pesticide frequencies revealed no significant disparities between the urban and rural zones [95]. Indeed, a preceding study in a nearby region detected residual levels of chlorpyrifos and monocrotophos in the water [96], with water and soil being other sources of contaminants for stingless-bee nests. Furthermore, it is imperative to implement comprehensive pesticide control strategies, encompassing both field and bee health measures. Additionally, research is necessary to substantiate the potential lethal threat to bees posed by exposure to banned pesticides.

The presence of polycyclic aromatic hydrocarbons (PAHs) in the environment is attributable to a variety of sources, including pyrogenic products resulting from the incomplete combustion of organic materials [97]. Petrogenic sources include petroleum by-products and coal distillation [93]. Biogenic sources are synthesized by biological entities during the slow biological conversion of organic materials [97]. For the honey of *M. marginata*, the results showed contaminants from pyrogenic sources. For the honey of species such as *M. bicolor*, *T. angustula*, and *S. postica*, the PAH contaminants were related to petrogenic sources. This study ranked PAH contamination using the ratio ΣCOMB/Σ16 PAHs. ΣCOMB is defined as the sum of Fluoranthene, Pyrene, Benz[*a*]anthracene, Chrysene, Benzo[*b*]fluoranthene, Benzo[*k*]fluoranthene, Benzo[*a*]pyrene, Indeno[1,2,3-*cd*]pyrene, and Benzo[*ghi*]perylene, while Σ16 PAHs represents the total of all 16 analyzed PAHs.

In this particular case, the location of the colonies in two different Brazilian locations was not shown to directly influence the results [60], and although the results showed two types of PAH sources, both come from anthropogenic activities.

### 4.3. Good Management Practices (GMPs) in Meliponiculture

It is recommended that good management practices in meliponiculture be adopted and applied to mitigate the risks of contamination, pollution, and pathogens. Certain stingless-bee species and the social wasp *Polybia scutellaris* have been observed robbing nests that still contain honey in cells or pots after the meliponiculture harvest season [39]. Therefore, it is recommended to conduct a thorough honey harvest and clean the nest, including its internal structures.

Feeding stingless bees with *A. mellifera* pollen and honey poses a health risk, as these products can carry diseases. Understanding the susceptibility of different bee species is crucial for assessing the impact of pathogens on their survival. Stingless-bee microbiomes may offer resistance to pathogens and diseases. Further research is recommended.

Another aspect of GMP in meliponiculture is bio-compartmentalization, a biosecurity procedure used to limit the spread of diseases among bees [98]. In practice, colonies should be well-spaced in open areas, maintaining at least 2 m between nests to prevent diseased bees from entering healthy colonies [99].

To combat/fight against diseases in stingless bees, queen replacement is practiced producing pathogen-resistant brood, along with selective breeding for hygienic behavior [100]. This selection must be carried out with the utmost diligence and in strict accordance with the guidelines established by the respective national health authorities.

Due to the lack of global regulations for stingless-bee honey as a food supplement [101], some studies, such as the one identifying PAHs in honey [60], have classified it as a special medical-purpose food. This classification allows for the comparison of contaminant concentrations and highlights potential human health risks associated with consuming PAH-contaminated honey.

However, at the regional level, there have been proposals for the establishment of standards with a view to their application in the regulation of stingless-bee honey. Such proposals have been made in the following countries: Bahia in Brazil (2014), Malaysia (2017), Tanzania (2017), Indonesia (2018), Argentina (2019), Australia (2024), and Thailand (2024). According to Vit et al. [102], now is the ideal time for stingless-bee honey regulations to be elevated to an international level, such as Codex Alimentarius. The first step must be the adoption of good management and sustainable practices in meliponiculture. Initiatives in this respect have been taken in Latin American countries, like Bolivia, Brazil, and Colombia. These countries have incorporated legal measures into their national laws intending to reduce risks to domesticated stingless bees [103].

### 4.4. One Health Approach

A “One Health” approach, which integrates the fields of environmental health, animal health, and human well-being, should be a critical component of stingless-bee management. This approach ensures sustainable meliponiculture practices by recognizing the interconnected nature of these fields. Stingless bees play a crucial role in maintaining biodiversity in tropical zones, enhancing crop yields, and producing honey with different applications in medicines, cosmetics, and foodstuffs. Therefore, their conservation is essential for ecosystem resilience and food security. Sustainable management strategies for these bees involve protecting natural habitats, minimizing pesticide exposure, and promoting diverse and native floral resources to support colony health.

The availability of plant species for stingless bees depends on land management. This management falls under political rulers’ jurisdiction. Environmental education programs, as well as reforestation, propagation, and seed rescue, are ways of working with communities directly [104]. Engaging local communities in educational and conservation efforts fosters resilience against climate change and habitat loss, ensuring the long-term viability of stingless-bee keeping.

Adopting biosecurity measures, along with responsible harvesting techniques and hygienic nest management, has been linked to a lower risk of pathogens spreading, benefiting stingless bees and their nest-by-product consumers. The implementation of additional preventive measures, such as the tracking and monitoring of the anthropogenic or environmental impacts on stingless bees, has the potential to be advantageous. This is because both the bees and the stingless-bee keeper can serve as an early warning system for environmental degradation and/or the presence of human health risks [105].

The care and management of stingless bees, as well as beekeeping, contributes to sustainability and promotes community living while stimulating local food production and a better understanding of ecosystems [106]. Moreover, this One Health cycle is completed with the human consumption of honey or pollen, or even the use of propolis and its derivatives in local medicine. Indeed, to ensure the quality of these products, the care of plants useful to bees should be the starting point.

## 5. Conclusions

A paucity of research exists on the pathogens associated with diseases affecting stingless bees. The reported effects include brood loss and annual death in a specific *Melipona* species.

Contaminants of anthropogenic origin have been found to accumulate in stingless-bee products at levels higher than those permitted in other matrices with which stingless-bee products can be compared.

The establishment of optimal practices and biosecurity measures in meliponiculture is imperative as an economic activity to support communities in tropical regions. This is crucial to mitigate risks to the survival and well-being of these species, which are confronted with natural enemies that are still being described.

The establishment of regional and global quality guidelines for stingless-bee by-products is imperative to ensure food security and product quality for both human consumption and other uses, such as nutraceuticals.

Further research is necessary to determine the impact of contaminants and pathogens on the physiology of stingless bees. It is imperative to avoid using honeybee references for lethal or sublethal doses of chemical contaminants in other bee species

## Figures and Tables

**Table 1:** Summary of pathogens (bacteria, fungi, and viruses) that are affecting stingless bees.

Stingless-Bee Species	Study Matrix	Disease/Pathogen	Study Prevalence	Detection Method	Habitat/Season	Country	Publication
*Melipona quadrifasciata*	Unhealthy and healthy adult individuals	Unknown annual colony collapse syndrome Disorder Firmicutes Group U (23%), Firmicutes Group Z (23%), and Acetobacteraceae (16%)	52 positives/76 samples = 0.68	PCR and Illumina MiSeq sequencing to analyze the variable region V1-V3 of the 16S rDNA gene	Managed nest Summer	Brazil	[50]
*Melipona marginata* *Melipona quadrifasciata* *Melipona mandacaia* *Melipona compressipes* *Melipona rufiventris* *Melipona mondury*	Brood, pollen, and honey	European Foulbrood *Melissococcus plutonius* Brood (66%), pollen (6%), honey (33%)	18 positives/30 mixed samples = 0.6	PCR and Sanger sequencing and fragment analysis applications, to analyze 16S rDNA gene	Managed nest distributed in an open and roofed area, in an orchard Spring	Brazil	[38]
*Tetragonula carbonaria* *Austroplebeia australis*	Workers and queen larvae, brood cell provisions, and honey pots	Bacterial brood disease *Lysinibacillus sphaericus* (Firmicutes, Bacillaceae) strains	Not specified	Characterization and pathogenicity by microbiology. PCR of the 16s rDNA gene, and cloning. Multilocus sequence typing (MLST) analysis	Managed colonies Summer	Australia	[37]
*Melipona subnitida*	Workers	Deformed wing virus variants DWV-A and DWV-C The average total viral loads per bee was 8.8 × 10^7^	21 stingless-bee positive/100 (10 pools of 10) = 0.21	RT-PCR of total RNA	Managed colonies Spring	Brazil	[34]
*Tetragonisca fiebrigi* *Scaptotrigona jujuyensis* *Tetragonisca angustula* *Melipona fasciculata* *Melipona quadrifasciata anthidioides* *Melipona marginata* *Melipona rufiventris* *Melipona mandacaia*	Adult individuals	Nosemosis *Nosema ceranae*	7 positives/8 species = 0.87	Duplex PCR of the 16S rRNA locus	Managed and wild colonies. Sampling over 5 years in Argentina, and one year in Brazil	Argentina and Brazil	[39]
*Melipona quadrifasciata*	Healthy and diseased forager bees	Tailed viruses (Caudoviricetes)	Not specified	DNA and RNA metagenomic	Not specified	Brazil	[51]
*Frieseomelitta varia* *Tetragonisca angustula* *Trigona spinipes* *Melipona quadrifasciata*	Adult individuals	Unknown annual syndrome *Pseudomonas* sp. *Sphingomonas* sp. *Escherichia coli* *Alcaligenes faecalis*	Not specified	PCR of the 16S rRNA gene (V3/V4 regions) and the MiSeq sequencing system	Managed colonies Spring–Summer	Brazil	[52]
*Nannotrigona testaceicornis* *Tetragonisca angustula* *Tetragona elongata*	Adult individuals	*Nosema ceranae* Acute bee paralysis virus (APBV) (10.8%) Deformed wing virus (DWV) (5.1%) Black queen cell virus (BQCV) (5.1%)	Histology detected spores in 100% stingless-bee bodies. Not detected in the midgut by PCR Viruses were found in 23.4% of stingless-bee samples.	Duplex PCR of 16S ribosomal gene RT-qPCR of mRNA from stingless bees	Managed nests Autumn–winter	Brazil	[53]

**Table 2:** Summary of contaminants found in stingless-bee by-products.

Stingless-Bee Species	Study Matrix	Contaminant [Min–Max]	Habitat/Season	Country	Publication
*Tetragonisca angustula*	Honey and pollen	As [1.70 ± 0.01–361.30 ± 18.88] μg kg^−1^ Cd [0.11 ± 0.01–1.64 ± 0.01] μg kg^−1^ In [0.08 ± 0.01–0.53 ± 0.29] μg kg^−1^ Pb [1.20 ± 0.01–463.31 ± 35.16] μg kg^−1^	Not specified	Brazil	[54]
*Melipona scutellaris*	Geopropolis	Cr [6.5–39.0] mg kg^−1^ Cu [1.9–8.4] mg kg^−1^ Mo [0.6–2.5] mg kg^−1^ Ni [0.8–6.8] mg kg^−1^ Pb [1.6–8.9] mg kg^−1^ Zn [1.2–21] mg kg^−1^ Cd [0.2–1.2] mg kg^−1^	Managed nests Urban environment Sampling over one year	Brazil	[55]
*Partamona helleri*	Larvae midguts	500 ng/bee of plastic microparticles of polystyrene (PS), and polyethylene terephthalate (PET) 10 μg/bee of nanoparticles of a metal oxide (titanium dioxide—TiO_2_)	Bioassay (laboratory conditions)*	Brazil	[56]
*Melipona quadrifasciata*	Honey	0.1 to 2.6 particles per honey mL of microplastics (primarily composed of polypropylene)	Managed nests Built-up and vegetated areas	Brazil	[57]
*Tetragonula carbonaria*	Bees, honey, and wax	As [12–140] μg kg^−1^ Pb [11–2050] μg kg^−1^ Mn [410–46,400] μg kg^−1^ Zn [490–73,000] μg kg^−1^	Managed nests Summer	Australia	[58]
*Scaptotrigona bipunctata* *Tetragonisca angustula* *Melipona quadrifasciata* *Tetragonisca weyrauchi* *Tetragona clavipes* *Scaptotrigona postica* *Melipona marginata*	Honey	Ca [0.70 ± 0.06–123.92 ± 1.49] μg g^−1^ Mn [0.66 ± 0.06–41.92 ± 4.67] μg g^−1^ Mg [1.60 ± 0.25–351.48 ± 9.58] μg g^−1^ Fe [13.04 ± 0.39–363.77 ± 6.41] μg g^−1^	Managed nests Atlantic Forest, and Amazon River Sampling over 4 years	Brazil	[59]
*Tetragonisca angustula* *Scaptotrigona depilis* *Scaptotrigona postica* *Melipona quadrifasciata* *Scaptotrigona bipunctata* *Melipona marginata* *Melipona bicolor*	Honey	1.4 to 23.3 μg kg^−1^ of polycyclic Aromatic Hydrocarbons (PAHs)	Managed nests Native forests and industrial areas Summer	Brazil	[60]
*Melipona quadrifasciata anthidioides*	Geopropolis	Al [20,414.40–36,911.1] mg kg^−1^ As [4.37] mg kg^−1^ Cr [17.41–38.07] mg kg^−1^ Ni [2.28–21.74] mg kg^−1^ Pb [3.45–8.55] mg kg^−1^ Sb [1.34–1.64] mg kg^−1^ Sn [4.92–16.14] mg kg^−1^	Managed nests Summer	Brazil	[61]
*Scaptotrigona mexicana*	Honey and pollen	Organochlorine compounds: Heptaclor [96.4–645.08] μg kg^−1^ γ-HCH [8.8–207.15] μg kg^−1^ α-HCH [3.8–4.79] μg kg^−1^ β-HCH [26.1–68.41] μg kg^−1^ p,p’-DDE [25.1–34.1] μg kg^−1^ Heptachlor epoxide [18.1–21.68] μg kg^−1^ α-Endosulfan [51–59.12] μg kg^−1^ p,p’-DDT [99–440.78] μg kg^−1^	Managed nests Sampling over one year	Mexico	[62]

Legend: Bioassay (laboratory conditions)*; Al = aluminum, As = arsenic, Ca = calcium, Cd = cadmium, Cr = chrome, Cu = copper, Fe = iron, In = indium, Mg = magnesium, Mn = manganese, Mo = molybdenum, Ni = nickel, Pb = lead, Sb = antimony, Sn = tin, Zn = zinc. HCH = hexachlorocyclohexane, DDT = dichlorodiphenyltrichloroethane, DDE = dichlorodiphenyldichloroethylene.

## Data Availability

Not applicable.
